# Controlled Multi-Dimensional Assembly of Calcium Carbonate Particles with Industrial By-Product Carbide Slag and CO_2_

**DOI:** 10.3390/nano15010016

**Published:** 2024-12-26

**Authors:** Yuke Shen, Xiaoli Jiang, Chengcai Tang, Wei Ma, Jianyu Cheng, Hongxu Wang, Hongyu Zhu, Lin Zhao, Yagang Zhang, Panfeng Zhao

**Affiliations:** School of Materials and Energy, University of Electronic Science and Technology of China, Chengdu 611731, China; 2021030902003@std.uestc.edu.cn (Y.S.); jiangxl@std.uestc.edu.cn (X.J.); 2021030902017@std.uestc.edu.cn (C.T.); 2021030902012@std.uestc.edu.cn (W.M.); 2023030902008@std.uestc.edu.cn (J.C.); 2023030902029@std.uestc.edu.cn (H.W.); zhy@uestc.edu.cn (H.Z.); zhaolin316@uestc.edu.cn (L.Z.)

**Keywords:** carbide slag, multi-dimensional assembly, calcium carbonate, energy conservation

## Abstract

The utilization of carbide slag, an industrial by-product, as a resource to prepare value-added products has a profound impact not only for sustainable synthesis and the circular economy but also for CO_2_ reduction. Herein, we report the very first example of the controlled multi-dimensional assembly of calcium carbonate particles at the micrometer scale with industrial by-product carbide slag and CO_2_. Calcium carbonate particles of distinctly different sizes, shapes, and morphologies are obtained by finely tuning the assembly conditions. This strategy yields diverse assembled structures, including simple cubic, mulberry-like assembled unit, stacked cubic polycrystalline, and rotated polycrystalline structures, using the same starting materials. This innovative approach not only highlights the adaptability and efficiency of utilizing industrial by-products via multi-dimensional assembly but also provides new insights into the potential applications of the resulting calcium carbonate.

## 1. Introduction

The rapid development of global industries and the increase in human activities have led to pressing issues related to carbon dioxide (CO_2_) emissions and industrial waste management. In this context, the effective utilization of industrial by-products to reduce environmental burdens, coupled with the efficient fixation and conversion of CO_2_, has drawn considerable attention in contemporary scientific research and industrial practices.

CaC_2_ + 2H_2_O → C_2_H_2_ + Ca(OH)_2_ (1)

Calcium carbide (CaC_2_) is the basic raw material of the chlor-alkali chemical industry, and about 70% of produced CaC_2_ is used for polyvinyl chloride (PVC) production. The main chemical reaction formula is as follows:

Calcium carbide slag (CS) is a by-product of the production of PVC through the method using CaC_2_, and its main component is calcium hydroxide. The calcium carbide approach is widely adopted in developing countries to prepare PVC. Approximately 20 tons of CS (with a water content of about 40–90%) is produced for every ton of PVC manufactured [1]. At present, the annual production of CS in China is around 40 million tons, and the accumulated inventory exceeds 100 million tons. Due to the strong alkalinity of Ca(OH)_2_, if not effectively treated, it not only occupies substantial land resources but also poses a risk of groundwater contamination [2], resulting in significant resource wastage and environmental challenges. In addition, CaC_2_ plays a crucial role as a key raw material in the production of acetylene (C_2_H_2_). The production of CaC_2_ typically involves a high-temperature reaction of calcium oxide (CaO) and coke in an electric arc furnace, represented by the following equation:

CaO + 3C → CaC_2_ + CO (2)

Meanwhile, CaO is produced via the calcination and decomposition of limestone (CaCO_3_) in a lime kiln at about 900 °C [3], which is a highly energy-consuming process. The pollutants produced in the process of CaC_2_ production are mainly gas pollutants and solid pollutants. Among them, the CaO small-particle raw materials (including powder) generated in the crushing and screening process become Ca(OH)_2_ after water absorption. Therefore, the efficient utilization of CS is imperative for the sustainable development and green production practices of the chlor-alkali industry.

The main component of CS is calcium hydroxide Ca(OH)_2_, along with small amounts of other inorganic compounds, such as metal oxides, phosphates, and sulfides, as well as organic impurities [4]. Studies have shown that CS may have potential for applications in wastewater treatment [5], gas adsorption [6,7,8], construction materials [9,10], and CO_2_ capture [11,12].

Micrometer-scale calcium carbonate has unique physical properties [13,14]. It can serve as a component of industrial products, such as filler materials in rubber [15], paper, ink [16], pigments [17], and coatings [18]. Its application range and effect vary depending on its particle size and morphology. In particular, nano calcium carbonate, because of its small particle size and high specific surface area, shows unique physical and chemical properties, such as good dispersion, high reactivity, and excellent filling properties. Additionally, CaCO_3_ possesses excellent biocompatibility and is an ideal material for biomedical applications such as antacid tablets, bone regeneration [19], and drug delivery systems [20]. At present, there are several approaches for preparing calcium carbonate particles. The ball-milling approach can obtain CaCO_3_ particles on the scale of hundreds of micrometers. Other approaches include the precipitation, CO_2_ bubbling, decomposition carbonization, and CO_2_ carbonization methods [21,22]. Prior research has focused on controlling various synthesis parameters [21,22,23].

More importantly, depending on its crystal form and shape, the application extent of CaCO_3_ in various fields also differs significantly. Particles of different forms exhibit substantial disparities in terms of their particle size, specific surface area, and fluidity. For instance, cubic CaCO_3_ is frequently employed as a filler in paints and inks [24,25]. Chain-like CaCO_3_ is applied in rubber and plastics due to its high reactivity and excellent reinforcing performance [26]. Porous CaCO_3_ is commonly utilized in biomedicine because of its unique core–shell structure [27,28,29]. Vaterite has the potential to be used as a controlled-release carrier because of its instability [30]. Thus, in-depth research on the evolution rules relating to crystal morphology is required to make better use of these forms.

According to the principles of green chemistry and sustainable development goals, the valorization of CS and CO_2_ through their co-utilization to produce nano calcium carbonate presents a promising approach to industrial by-product transformation and greenhouse gas mitigation. The proposed reaction leverages CS (the main component of which is Ca(OH)_2_) as a calcium source, reacting with industrial flue gas or purified CO_2_ to form calcium carbonate:

Ca(OH)_2_ + CO_2_ → CaCO_3_ + H_2_O (3)

This process not only sequesters CO_2_ but also transforms a challenging industrial waste product into a high-value-added material, epitomizing a “waste-to-wealth” strategy. Although the synthesis of nano–micro calcium carbonate mediated by additive engineering has been systematically explored [31], the examples of controlled multi-dimensional assembly of calcium carbonate directly from CS and CO_2_ are quite few.

Recently, supramolecular chemistry has evolved with the characteristics of dynamic recognition, stimulus response, adaptiveness, specific recognition, and selectivity. Well-defined structures for specific functions are determined through precise recognition at the molecular level [32]. Functionally oriented molecular assembly has emerged as a powerful tool to achieve this goal [33]. However, most of the assembled supramolecular architectures are organic systems. Precise control over size and morphology in an inorganic system is extremely challenging and rare.

It is reported that there are various factors affecting the assembly process of CaCO_3_. Factors such as additives, the raw material concentration [34], the reaction pH [1,21,35], the flow rate, and impurities [36] all contribute towards the particle size and distribution. This study focuses on the effects of impurities, additives, and the CO_2_ flow rate in CS on CaCO_3_ assembly. The morphology and size of crystals can be micro-controlled by adding different kinds and amounts of additives, through molecule matching and electrostatic interactions [31]. Impurities act similarly to additives, allowing the growth of calcium carbonate into specific crystal types and shapes through ion adsorption and by occupying low-surface-energy crystal surfaces [1,36]. In the CO_2_ carbonization method, with an increase in the CO_2_ flow rate, on the one hand, the saturation of calcium carbonate in the solution increases, and the nucleation rate of calcium carbonate increases. On the other hand, the continuous flow of CO_2_ interferes with the growth of carbonate calcium and thereby affects the size and shape of the crystal [37].

**Figure 1:**
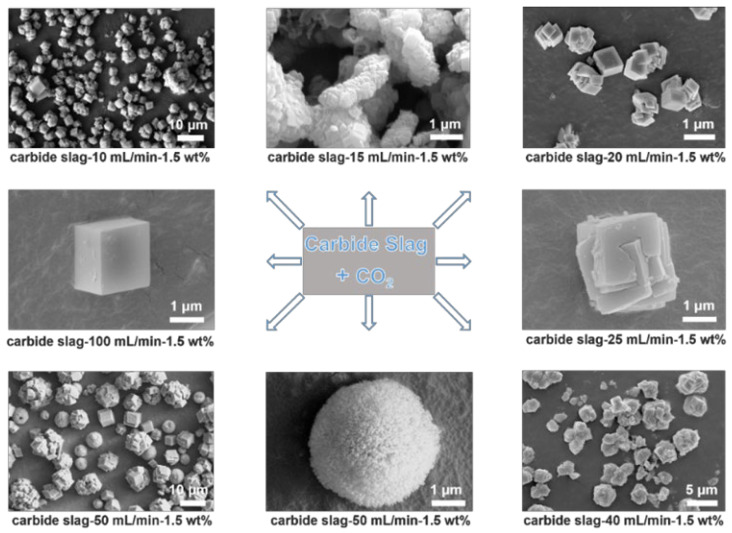
SEM images of carbonation products (CaCO_3_) obtained from CS and CO_2_ under different reaction conditions.

In this context, we report the very first example of a controlled multi-dimensional assembly of calcium carbonate particles at the micrometer scale using industrial by-product CS and CO_2_. More importantly, the morphology and size of the calcium carbonate particles can be tailored by tuning the reaction conditions (such as the temperature, pH, and additives), enabling their multi-dimensional assembly at the micro–nanoscale. Notably, we found that by meticulously controlling the reaction conditions, calcium carbonate particles of distinctly different sizes, shapes, and morphologies can be obtained using the same raw materials. The nucleation, growth, and unit cell packing of calcium carbonate are regulated by controlling the concentration of palmitic acid (used as an additive) and the flow rate of CO_2_. In addition, due to the influence of impurities in CS on crystal growth and assembly, and its interplay with the CO_2_ flow rate, diverse assembled structures, including simple cubic, mulberry-like assembled unit, stacked cubic polycrystalline, and rotated polycrystalline structures, are obtained (Figure 1).

## 2. Materials and Methods

Carbide slag (CS) was obtained from Wanhua Chemical (Yantai, China) Chlor-alkali Thermoelectric Power Co., Ltd. CO_2_ with a purity of 99.999% was purchased from Dalian Special Gases Co., Ltd (Dalian, China). Calcium hydroxide (Ca(OH)_2_, AR grade) was purchased from Chengdu Jinshan Chemical Reagent Co., Ltd. (Chengdu, China). Palmitic acid (AR grade) was purchased from Heowns Biochem Technologies, LLC (Tianjin, China). Anhydrous ethanol (AR grade) was purchased from Shanghai Titan Scientific Co., Ltd (Shanghai, China).

The process is as follows: Firstly, dissolve excessive CS/calcium hydroxide in 100 mL of deionized water. Filter the solution to obtain a saturated solution of CS/calcium hydroxide. Meanwhile, prepare an anhydrous ethanol solution of palmitic acid (0.1 M). Subsequently, add 100 mL of the saturated solution of CS/calcium hydroxide and a certain quantity of additives into a beaker to form a mixed solution. Then, place the solution on a stirring platform, with the stirring speed set at 300 r/min and the temperature maintained at 30 °C. During the stirring process, immerse an aeration stone connected to a CO_2_ gas pipeline into the solution, and commence the introduction of CO_2_ gas at different flow rates until the pH reaches 7. Subsequently, filter the mixture in the beaker and rinse repeatedly with water to collect the solid powder. Finally, dry the solid powder in an oven at 60 °C for 10 min to obtain nano–micro CaCO_3_. Herein, the obtained materials are denoted by CS/Ca(OH)_2_-xwt%-y (x = 1.5, 3.0, and 4.5, which represents the percentage by weight of the additive incorporated in relation to the weight of the expected product, and y = 10–150, corresponding to the CO_2_ flow rate of 10–150 mL/min). The specific amounts of additives and the specific flow rates of CO_2_ are presented in Table 1.

## 3. Results

CaCO_3_ exists in three crystalline forms: calcite, aragonite, and vaterite. Among these, calcite is the most thermodynamically stable, while both vaterite and aragonite are unstable and prone to polymorphic transformation [14]. The formation of crystals involves two stages—nucleation and crystal growth—which can occur through two growth modes: “equilibrium” and “growth.” The “equilibrium” mode refers to a thermodynamic analysis where the shape of a particle of a given volume tends to evolve towards the crystal morphology that minimizes the surface energy, ultimately resulting in an equilibrium shape. Typically, under natural conditions, the crystal shape corresponds to the equilibrium shape. The “growth” mode, on the other hand, is based on the kinetic parameters of the crystal faces; during the crystal growth phase, faster-growing faces gradually disappear, leaving behind the slower-growing faces that define the crystal morphology. In this study, we controlled the reaction rates during different crystallization stages of calcium carbonate through the use of process parameters (the CO_2_ gas flow rate and liquid phase concentration) to ultimately achieve control over the crystal size and morphology. Crystal morphology control agents are substances added during the crystal growth process that can adsorb onto the microcrystalline surfaces or act as nuclei and templates in crystal formation, thereby influencing the growth rates of the crystal faces.

**Figure 2:**
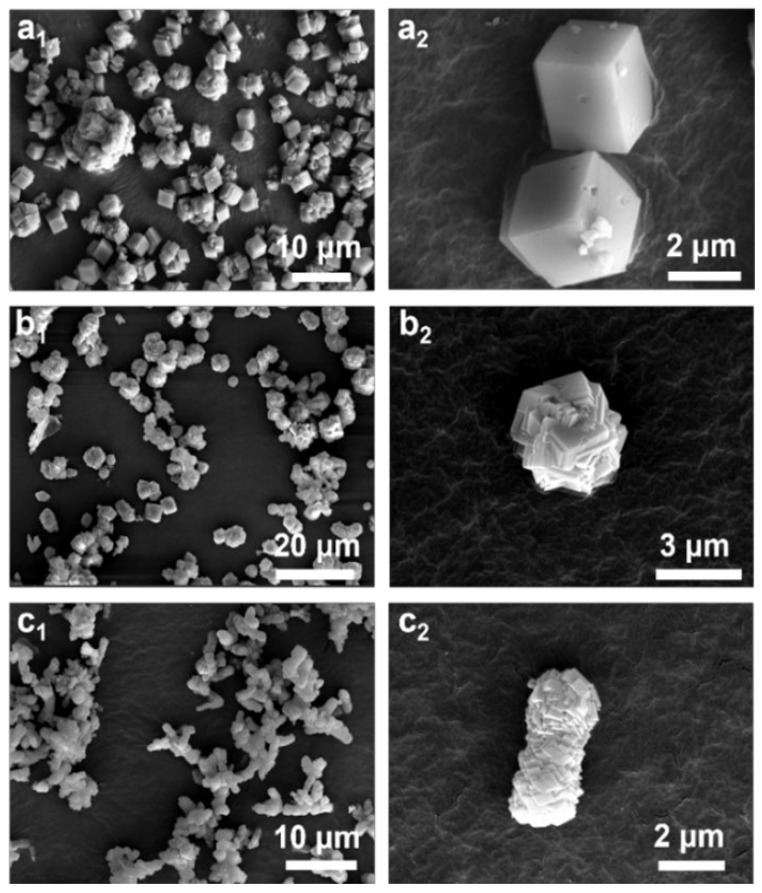
SEM images of calcium carbonate with different amounts of palmitic acid additive: (**a_1_**,**a_2_**) 1.5 wt% (CS-1.5wt%-10); (**b_1_**,**b_2_**) 3 wt% (CS-3.0wt%-10); (**c_1_**,**c_2_**) 4.5 wt% (CS-4.5wt%-10).

Palmitic acid, a saturated fatty acid, is commonly employed as a surfactant. In the synthesis of calcium carbonate, a carbon chain of a certain length forms a certain shape of micelle in the Ca(OH)_2_ slurry, and this micelle acts as a template for the formation of CaCO_3_ particles. However, the addition of palmitic acid at different concentrations changes the contact mode of the micelles, thus determining the aggregation form of CaCO_3_ particles [38,39]. To explore the effect of palmitic acid on the morphology of calcium carbonate obtained using CS and CO_2_, field emission scanning electron microscopy (FE-SEM) was conducted to characterize the calcium carbonate samples synthesized using varying concentrations of palmitic acid. The results showed that at a palmitic acid concentration of 1.5 wt%, the synthesized calcium carbonate exhibited a simple cubic morphology with dimensions of approximately 2 μm, along with stacked cubes and a small proportion of rotated polyhedral particles (Figure 2(a_1_,a_2_)). These particles were well dispersed. Specifically, palmitic acid molecules in solution adsorb onto specific crystal faces of CaCO_3_, thereby reducing the surface energy of those faces. Due to these changes in the surface energy of different crystal faces, the crystal growth rate is either suppressed or guided, leading to the formation of specific and stable crystal morphologies (e.g., cubic calcite crystals). At low concentrations of palmitic acid, the growth direction of the crystals is not significantly inhibited, and the commonly observed calcite morphology may form.

Palmitic acid primarily functions by forming individual micelles and limiting growth along certain crystal faces, resulting in relatively uniform growth in all directions and thus favoring the formation of regular cubic structures. With an increase in the palmitic acid concentration to 3 wt%, the calcium carbonate particles tended to assemble into spherical aggregates with a diameter of approximately 3 μm, composed of rotated polycrystalline subunits (Figure 2(b_1_,b_2_)). This morphological change can be explained by the aggregation of multiple micelles, inhibiting the identical replication of unit cells in one direction and promoting rotational stacking of the unit cells [39] This led to the formation of rotated polycrystalline stacked structures. As shown in Figure 2(c_1_,c_2_), a further increase in the palmitic acid concentration to 4 wt% resulted in the formation of mulberry-like aggregates approximately 5 μm in length, composed of stacked nanosheets. These aggregates exhibited a uniform morphology and size distribution. Under these conditions, the nucleation rate increased significantly, leading to the formation of smaller crystalline units in the form of nanosheets. These nanosheets subsequently assembled and stacked to form more complex three-dimensional mulberry-like structures.

This indicates that varying the concentration of palmitic acid can effectively control the particle morphology. The progression from simple cubes to rotated polycrystals and, finally, to mulberry-like structures composed of stacked nanosheets illustrates the ability of palmitic acid to regulate the anisotropic growth of crystals. This morphological evolution can be explained by the interaction of palmitic acid molecules with the aqueous phase or ions through their long-chain fatty acid groups, forming micelles and leading to adsorption on the surface of calcium carbonate particles. This surface adsorption reduces the surface energy of the particles, promotes nucleation, and consequently alters the morphology of the particles [38,39].

**Figure 3:**
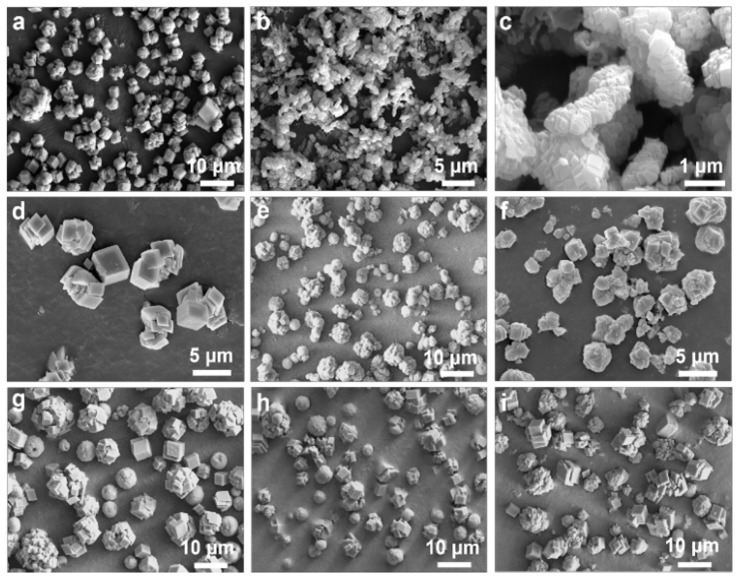
SEM images of CaCO_3_ prepared with CS under the conditions of a 1.5wt% palmitic acid additive and different CO_2_ injection rates: (**a**) 10 mL/min, (**b**,**c**) 15 mL/min, (**d**) 20 mL/min, (**e**) 25 mL/min, (**f**) 40 mL/min, (**g**) 50 mL/min, (**h**) 100 mL/min, and (**i**) 150 mL/min.

The influence of the CO_2_ flow rate on the morphology of calcium carbonate was investigated. We conducted a series of experiments while maintaining a constant addition rate of 1.5 wt% palmitic acid and keeping the other parameters the same. From Figure 3a, it can be observed that at lower CO_2_ flow rates of CS-1.5wt%-10, the predominant morphology of the CaCO_3_ was simple cubic structures. This result can be attributed to the lower dissolution rate of CO_2_ in the solution, resulting in a slower nucleation rate. Consequently, the crystals had ample time for identical unit cell packing in all directions, thereby forming regular cubic shapes. Furthermore, at lower CO_2_ flow rates, palmitic acid likely acts to stabilize the crystal surfaces, inhibiting excessive growth and ensuring the regularity of the crystal shapes [38,40]. For CS-1.5wt%-15 (Figure 3b,c), the CO_2_ dissolution rate was elevated, accelerating the nucleation rate, which, in turn, promoted rapid crystal growth.

With a further increase in the CO_2_ flow rate to 20 mL/min (CS-1.5wt%-20), the collision frequency between Ca^2^⁺ and CO_3_^2^⁻ ions was elevated, which enhanced the nucleation rate and resulted in rapid crystal growth and stacking (Figure 3d). For CS-1.5wt%-25 and CS-1.5wt%-40, the nucleation rate increased even further, resulting in rapid crystal growth with stacked rotations. During this stage, palmitic acid may modulate the direction and rate of crystal growth, leading to the formation of polycrystalline rotational structures (Figure 3e,f). With an increase in the CO_2_ injection rate to 50 and 100 mL/min (CS-1.5wt%-50 and CS-1.5wt%-100), there was a significant rise in the CO_2_ concentration, resulting in a very high nucleation rate. This caused rapid crystal growth and induced the formation of distinctly different shapes and morphologies (Figure 3g,h). It is postulated that palmitic acid simultaneously inhibited and promoted growth in different directions during this stage, leading to the coexistence of simple cubic, polycrystalline, rotational, and spherical morphologies. As shown in Figure 3i, when the CO_2_ flow rate was increased to 150 mL/min (CS-1.5wt%-150), the multi-dimensional assembly of CaCO_3_ occurred at the same time. Noticeably, the generation of bubbles and aggregation of particles negatively impacted the particles’ uniformity.

The CO_2_ flow rate directly influences the supersaturation, nucleation rate, and crystal growth rate of calcium carbonate by controlling the supply rate of CO_2_ in the solution. First, CO_2_ dissolves in water to form carbonic acid (H_2_CO_3_), which subsequently dissociates into H^+^ and CO_3_^2−^ ions. A higher CO_2_ flow rate results in faster generation of carbonic acid in the solution, thereby increasing the supersaturation of calcium carbonate and promoting an enhanced nucleation rate. At low flow rates, the increase in supersaturation is gradual, leading to a reduced nucleation rate, allowing the crystals to grow for a longer duration and resulting in larger, more regular, and thermodynamically stable crystals (such as typical calcite CaCO_3_). Conversely, at high CO_2_ flow rates, the rapid formation of high supersaturation occurs, leading to the rapid generation of numerous nuclei, which can easily result in spherical or granular structures and may induce the formation of metastable phases (such as spherical vaterite) [41,42].

In other studies, researchers also investigated the effect of the CO_2_ flow rate on crystal morphology. Pan et al. [36] investigated the influence of the CO_2_ flow rate on carbonation production. They found that as the CO_2_ flow rate increased, the calcite morphology changed from rod-like to irregular particles, and they suggested that the bubbles generated by the higher CO_2_ flow rate accelerated collisions and contact between the particles.

**Figure 4:**
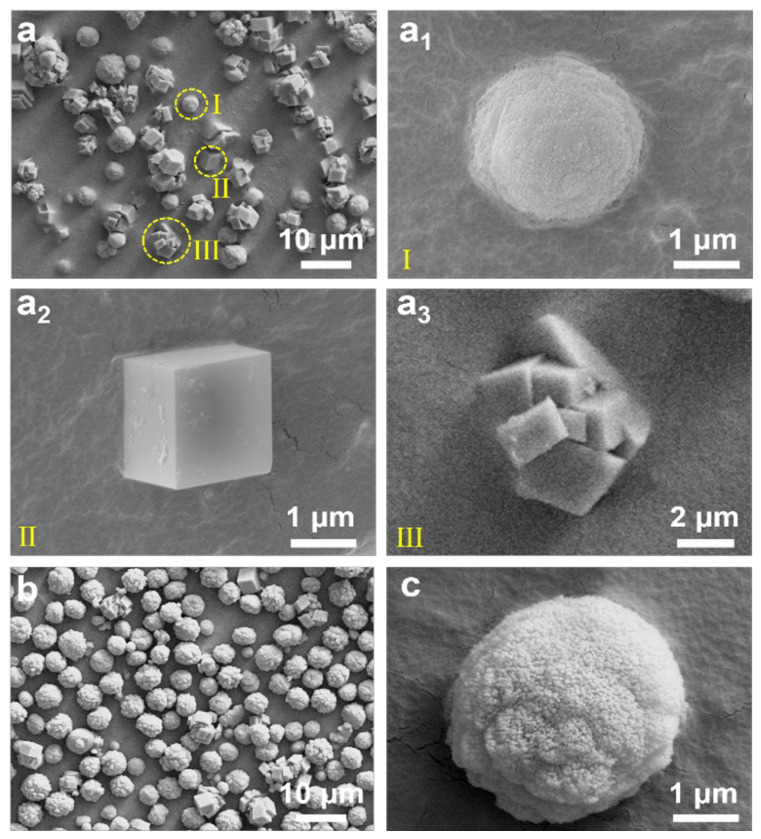
SEM images of (**a**) CaCO_3_ prepared with CS as the raw material under conditions of 1.5 wt% palmitic acid and a CO_2_ flow rate of 100 mL/min (CS-1.5wt%-100) and (**a_1_**–**a_3_**) corresponding high-magnification images of the observed morphologies. SEM images at (**b**) low magnification and (**c**) high magnification of CaCO_3_ prepared with filtered calcium hydroxide as the raw material under the conditions of a 1.5wt% palmitic acid additive and a CO_2_ flow rate of 100 mL/min (Ca(OH)_2_-1.5wt%-100).

CS is a by-product of the reaction between calcium carbide (CaC_2_) and water, and its main component is Ca(OH)_2_. It contains various impurities, such as silicon dioxide (SiO_2_), aluminum oxide (Al_2_O_3_), and iron oxide (Fe_2_O_3_) [36]. To investigate the influence of impurities in CS on the morphology of CaCO_3_, a control carbonization reaction of CS was carried out using a filtrate obtained by dissolving an excessive amount of analytical-grade Ca(OH)_2_, under the same conditions with regard to palmitic acid addition and the CO_2_ aeration rate. As shown in Figure 4a, when CS was used as the raw material (CS-1.5wt%-100), the obtained CaCO_3_ consisted of relatively dense spherical structures composed of small particles (Figure 4(a_1_)), along with simple cubic (Figure 4(a_2_)) and rotating polycrystal (Figure 4(a_3_)) morphologies. This phenomenon can be attributed to the fact that the impurities in CS act as heterogeneous nucleation agents during the nucleation and crystallization processes, providing additional nucleation sites and promoting the rapid nucleation of CaCO_3_ at different locations, resulting in the coexistence of crystals with different morphologies [36]. Moreover, the presence of impurities leads to an uneven distribution of Ca^2+^ and CO_3_^2−^ in the solution, resulting in higher local concentrations of Ca^2+^; this triggers rapid nucleation and growth, leading to the formation of polycrystalline rotational packing and dense spherical structures [43].

When calcium hydroxide bought from a reagent company was directly used as the control raw material (Ca(OH)_2_-1.5wt%-100), the obtained CaCO_3_ particles had a spherical structure with small pores on their surface and a relatively uniform size distribution (Figure 4b,c). This is because pure calcium hydroxide provides a uniform distribution of Ca^2+^ in the calcium hydroxide solution, resulting in a uniform nucleation process and the formation of small and evenly dispersed particles. These particles aggregate during the growth of CaCO_3_, forming spherical structures. Under high CO_2_ aeration rates, the rapid reaction of CO_2_ leads to the rapid combination of Ca^2^⁺ and CO_3_^2−^ to form CaCO_3_, and the generated gas may help to form tiny pores on the surface of the assembled particles [44].

**Figure 5:**
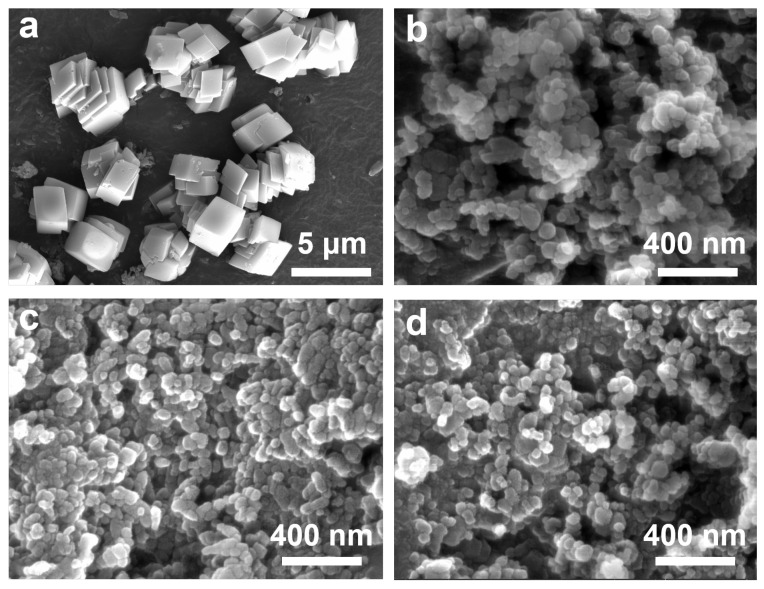
SEM images of CaCO_3_ prepared with unfiltered calcium hydroxide solution as the raw material under the conditions of a 1.5wt% palmitic acid additive and CO_2_ injection rates of (**a**) 10 mL/min, (**b**) 25 mL/min, (**c**) 50 mL/min, and (**d**) 100 mL/min.

When an unfiltered calcium hydroxide solution was used as the raw material, the morphologies of the calcium carbonate (CaCO_3_) particles at low CO_2_ flow rates were predominantly simple cubic and polycrystalline (Figure 5a). As the aeration rate increased, the CaCO_3_ transitioned to an aggregated nanoparticle morphology (Figure 5b). Further increases in the CO_2_ aeration rate did not trigger significant morphological changes (Figure 5c,d).

A plausible explanation for this is that when an excessive amount of calcium hydroxide is used as a starting material without filtration, dissolved calcium hydroxide is consumed in a reaction with CO_2_, and excessive calcium hydroxide continues to dissolve. The portion of Ca(OH)_2_ that continues to dissolve acts as an impurity relative to the already-formed CaCO_3_, potentially encapsulating the surface of the newly formed CaCO_3_ and inhibiting crystal unit replication and packing, thus favoring the formation of smaller-sized CaCO_3_ at the nanometer scale. In contrast, when the calcium hydroxide solution is filtered, there is no substantial re-dissolution of calcium hydroxide to encapsulate the crystal surface, allowing the crystals to continue growing, thus enabling the formation of larger single crystals.

Compared with other approaches, the preparation route in this study is simple, with lower energy consumption, and the utilization of CS waste is more direct. Notably, our method allows for control of the CaCO_3_ particle morphology and size through varying the amount of additives and adjusting the CO_2_ flow rate. The effects of different methods on the morphology of CaCO_3_ particles are summarized in Table 2.

## 4. Conclusions

In conclusion, micrometer-scale assembled calcium carbonate particles with distinctly different morphological features were successfully prepared using industrial by-product CS and CO_2_. The controlled multi-dimensional assembly of calcium carbonate particles was achieved by controlling the reaction conditions, including the amount of palmitic acid added, the CO_2_ flow rate, and the type of impurities. The findings indicate that the nucleation and growth of calcium carbonate were significantly influenced via the regulation of the palmitic acid concentration and CO_2_ aeration rate. This example demonstrates the power and versatility of molecular assembly in creating new structures, highlighting their potential for sustainable synthesis and contribution to a circular economy.

## Figures and Tables

**Table 1:** The amounts of additives, different sources of Ca(OH)_2_, and flow rates of CO_2_.

Name	Ingredients	n_(additives)_ (mmol)	Flow Rates of CO_2_ (mL/min)
CS-1.5wt%-10	CS	0.0032	10
CS-3.0wt%-10	CS	0.0097	10
CS-4.5wt%-10	CS	0.0129	10
CS-1.5wt%-15	CS	0.0032	15
CS-1.5wt%-20	CS	0.0032	20
CS-1.5wt%-25	CS	0.0032	25
CS-1.5wt%-40	CS	0.0032	40
CS-1.5wt%-50	CS	0.0032	50
CS-1.5wt%-100	CS	0.0032	100
CS-1.5wt%-150	CS	0.0032	150
Ca(OH)_2_-1.5wt%-100	Ca(OH)_2_	0.0034	100

**Table 2:** Effects of different methods on the morphology of CaCO_3_ particles.

Methods	Raw Materials	Conditions	Morphology	Reference
CO_2_ bubbling	CS + CO_2_	CO_2_: 10, 15, 20, 25, 40, 50, 100, 150 mL/min Palmitic acid amounts: 1.5, 3.0, 4.5wt%	Spherical vaterite +cube calcite + mulberry calcite	This work
	Ca(OH)_2_ + CO_2_	CO_2_: 100 mL/min Palmitic acid amounts: 1.5wt%	Spherical vaterite +cube calcite	
CO_2_ bubbling	CS + CO_2_	Solid/liquid ratio: from1:100 to 10:100, CO_2_: 200 mL/min	Rod-shaped calcite	[36]
	Ca(OH)_2_ + CO_2_	Solid/liquid ratio: 10:100, CO_2_: 200, 300, 500 mL/min	Spherical calcite	
CO_2_ bubbling ((NH_4_)_2_SO_4_)	CS + CO_2_	CO_2_: 500 mL/min NH_4_^+^/Ca^2+^ = 2.4	Spherical vaterite +cube calcite	[23]
CO_2_ bubbling (NH_4_Cl)	CS + CO_2_	CO_2_: 60 mL/min Time: 60min	Spherical vaterite +cube calcite	[37]
CO_2_ bubbling (sodium citrate)	CS + CO_2_	CO_2_: 0.5~1.5 L/min Terminal pH: 11	Calcite micro-/nanorods	[1]
CO_2_ bubbling	CS + CO_2_	Alkaline system: solid/liquid ratio: 1:100 Acidic system: solid/liquid ratio: 5:100, 10:100	Cubic calcite +spindle calcite	[21]

## Data Availability

The datasets presented in this study are available on request to the authors.
